# Investigation of Swedish cases reveals an outbreak of cryptosporidiosis at a Norwegian hotel with possible links to in-house water systems

**DOI:** 10.1186/1471-2334-8-152

**Published:** 2008-11-01

**Authors:** Agnes Hajdu, Line Vold, Torild A Østmo, Anna Helleve, Sigrid R Helgebostad, Truls Krogh, Lucy Robertson, Birgitta de Jong, Karin Nygård

**Affiliations:** 1 Department of Infectious Disease Epidemiology, Norwegian Institute of Public Health, Oslo, Norway; 2 European Programme for Intervention Epidemiology Training (EPIET), Swedish Institute for Infectious Disease Control, Solna, Sweden; 3 District Office for Asker and Bærum, Norwegian Food Safety Authority, Sandvika, Norway; 4 Municipal Health Authority, Asker, Norway; 5 Department of Water Hygiene, Norwegian Institute of Public Health, Oslo, Norway; 6 Department of Food Safety and Infection Biology, Norwegian School of Veterinary Science, Oslo, Norway; 7 Department of Communicable Disease Control and Prevention, Stockholm County Council, Stockholm, Sweden

## Abstract

**Background:**

In March 2007, the Norwegian Institute of Public Health was notified of Swedish individuals diagnosed with cryptosporidiosis after staying at a Norwegian hotel. In Norway, cryptosporidiosis is not reportable, and human infections are rarely diagnosed.

**Methods:**

A questionnaire on illness and exposure history was e-mailed to seven organised groups who had visited the hotel in March. Cases were defined as persons with diarrhoea for more than two days or laboratory-confirmed cryptosporidiosis during or within two weeks of the hotel visit. The risk factor analysis was restricted to two groups with the highest attack rates (AR) and same hotel stay period. Local food safety authorities conducted environmental investigations.

**Results:**

In total, 25 diarrhoeal cases (10 laboratory-confirmed) were identified among 89 respondents. Although environmental samples were negative, epidemiological data suggest an association with in-house water consumption. In one group, the AR was higher amongst consumers of water from hotel dispenser (relative risk [RR] = 3.0; 95% confidence interval [CI]: 0.9–9.8), tap water (RR = 2.3; CI: 0.9–5.8), and lower amongst commercial bottled water drinkers (RR = 0.6; CI: 0.4–1.0). Consumption of ice cubes was a risk-factor (RR = 7.1; CI: 1.1–45.7) in the two groups combined.

**Conclusion:**

This outbreak would probably have remained undetected without the alert from Swedish health authorities, illustrating the difficulties in outbreak detection due to low health care seeking behaviour for diarrhoea and limited parasite diagnostics in Norway. Awareness of cryptosporidiosis should be raised amongst Norwegian medical personnel to improve case and outbreak detection, and possible risks related to in-house water systems should be assessed.

## Background

*Cryptosporidium *is recognised as an emerging pathogen and a common cause of diarrhoeal disease worldwide [1]. The gastrointestinal disease is usually mild and self-limiting, but potentially severe, and can be life-threatening in persons with weaker immune systems, such as infants, elderly, and immuno-compromised persons. In the last three decades, a large number of waterborne outbreaks of cryptosporidiosis have been reported in North America and in Europe [2]. The parasite is widespread in untreated surface water, highly resistant to chemical disinfectants commonly used in drinking water treatment, and has a low infectious dose. The species causing most cases of human cryptosporidiosis in Europe are *C. parvum *and *C. hominis *[3].

In Norway (population 4.7 million), cryptosporidiosis is not a notifiable infection. A laboratory survey showed that cryptosporidiosis is rarely diagnosed; between 1998 and 2002, less than three cases were diagnosed annually in five of the 14 medical microbiology laboratories with diagnostic routines for this pathogen [4]. Laboratories rarely examine human faecal samples for *Cryptosporidium *oocysts [4], nevertheless enhanced surveillance during a large waterborne giardiasis outbreak in 2004 found that several diarrheic patients had non-identical *C. parvum *infections [5]. The only documented human outbreak of cryptosporidiosis in Norway was associated with contact with calves at a dairy farm [6].

On 28 March 2007, the Norwegian Institute of Public Health (NIPH) was notified that several Swedish persons had developed a diarrhoeal illness following a stay at a hotel in Norway in mid-March. On 30 March, the reporting Swedish health authority confirmed that *Cryptosporidium *oocysts were detected by standard microscopy with Ziehl-Nielsen staining in stool samples from four of these individuals. At that time, the NIPH had not received any alert of a suspected gastrointestinal outbreak from the area. The local food safety authority and the municipal health authorities were informed and contacted the facility, a conference hotel with 109 guest rooms and 23 meeting rooms. An investigation was initiated to confirm the outbreak, assess its extent, and identify the vehicle of transmission in order to implement control measures. In this report, we describe the epidemiological and environmental investigations of this outbreak.

## Methods

### Epidemiological investigation

According to information from the hotel, eleven organised groups of companies and professional societies stayed at the hotel during the period 15–25 March 2007. For each group, we informed contact persons about the outbreak alert and asked whether they knew of any individuals in their group who had suffered from gastrointestinal illness during or after the stay in the hotel. Seven groups (consisting of 158 people) were included in further epidemiological investigations. Four groups either refused to participate or were excluded due to practical difficulties in accessing group members. According to information from the contact persons, amongst these four groups, none of the members presented with gastrointestinal symptoms during or after their hotel stay. Information about the meals served and other foods and drinks available at the hotel in March was provided by the hotel administration.

#### Descriptive study

We conducted a retrospective cohort study among the seven groups. A case was defined as a person who had stayed at hotel X between 10 and 25 March 2007, and had either self-reported diarrhoea (two or more loose stools per day) for more than two days, or had laboratory-confirmed cryptosporidiosis during or within three weeks of their stay at the hotel. A web-based questionnaire (QuestBack) was developed which focused on demographic data, clinical symptoms, detailed exposure-history to food and drinks with special regard to different types of water consumed at various points at the hotel (e.g. drinking water from the tap, from dispensers, from jugs in meeting room, commercial bottled water, purified water bottled in the hotel, ice cubes) and use of the swimming pool. The link to the web-questionnaire was sent to the contact persons who were asked to forward it to every group member.

#### Analytical study

Based on the results of the descriptive study, the risk factor analysis was restricted to those two groups (groups C and D) which had high attack rates or laboratory-confirmed cases and which had stayed at the hotel during the same weekend, 16–18 March 2007.

#### Data management

Questionnaire data were exported from the QuestBack database, and were analysed in Episheet (Rothman, K., version 26 June 2004) and STATA V9.1 (STATA Corporation, College Station, TX, USA). In the descriptive study, we calculated group-specific attack rates and the proportion of people exposed to a given food or drink. In the analytical study, bivariable analysis of each exposure was conducted in group C separately, as well as in groups C and D merged. The results are presented as risk ratios (RR) with 95% confidence intervals (CI) and two-tailed Fisher's exact p values.

Investigations of outbreaks are regulated by the Infectious Disease Control Act and regulations in Norway. An investigation to identify the source and implement control measures is considered as an urgent public health task. Due to this, such investigations are exempted from the requirement of approval from ethical review board. This is in agreement with the International Guidelines for Ethical Review of Epidemiological Studies by the Council for International Organisations of Medical Sciences (CIOMS) (1991). Additionally, no oral or written informed consent was required in this case, since the individuals could choose whether or not to answer the questionnaire after being given information on the purpose and scope of the investigation in the preamble, and had the option of remaining anonymous.

### Clinical microbiological investigation

Stool samples were examined by standard microscopy with Ziehl-Nielsen staining for presence of *Cryptosporidium *oocysts. Molecular typing was performed in Sweden using PCR-RFLP-analysis of the *Cryptosporidium *oocyst wall protein [7] and the 18S rRNA gene [8].

### Environmental investigation

On 29 March 2007, the day after the outbreak alert, the local food safety authority inspected the hotel, in particular the kitchen and water appliances, and interviewed hotel personnel. One food sample of raw beef carpaccio was sent for laboratory analysis for common bacterial enteropathogens. On 2 April, nine 10-litre water samples of raw and treated water were collected at the municipal water plant and from different points (including water dispensers) in the hotel and sent for analysis for parasites at the Swedish Institute for Infectious Disease Control. The water samples were tested using the ISO 15553 method [9]. The samples were concentrated by membrane filtration using polycarbonate filter 293 mm diameter, 2 μm pore size (Poretics # 20CP29320).

On 22 May, the cellulose filter (Cuno Micro Klean II; G78 B2) of the ice machine placed in the small supplementary kitchen used only for serving water to meeting rooms (hereafter referred to as "meeting rooms' kitchen") was examined for *Cryptosporidium *oocysts at the Norwegian School of Veterinary Sciences (NVH). The outer layer of the filter was removed and cut into pieces of a maximum dimension of 2 cm^2^. These underwent three washing and one sonification procedure using detergent-based buffer that contains salts, detergents, and Antifoam A [10] and distilled water (ratio 1:4). The combined collected fluid was concentrated by centrifugation (10 min at 1000 × *g*) and resulted in a pellet of approximately 20 to 30 ml, of which a relatively small percentage was due to the filter matrix itself, being readily distinguishable by the larger particle size. The standard water immunomagnetic separation (IMS) procedure (GC-Combo; Dynal Biotech, Invitrogen) was conducted in duplicate on 10 ml volumes each containing not more than 0.5 ml pellet (i.e. a maximum of 5% of the filter was analysed). The final volumes from each IMS were dried and fixed to slides before staining with monoclonal antibody against both *Giardia *cysts and *Cryptosporidium *oocysts (Aqua-Glo; Waterborne Inc., New Orleans) and 4'6 diamidino-2-phenyl indole, then screened by fluorescent microscopy using the appropriate filter sets.

Investigation of possible leakage or other sources of water contamination in the local water supply area was conducted by the municipal waterworks and past records of water quality tests were reviewed. As part of another investigation, further raw and treated water samples (10-litre volumes) were collected on four separate occasions in mid-June in the same area and analysed at the NVH by US EPA (United States, Environmental Protection Agency) Method 1623, using membrane filtration as the first step in the procedure.

## Results

### Epidemiological investigation

#### Descriptive study

We received 89 answers to the electronic questionnaire which corresponds to a 56% response rate, assuming that all 158 persons received the link from the contact persons of their respective groups.

In total, 25 cases were identified in three groups (groups B, C, and D), with 22 of the cases in group C (attack rate 67%) which included both Norwegians and Swedes (table 1). Nine laboratory-confirmed cases were identified in groups C and one in group D; the two groups had the same dates of visit to the hotel. Of all the respondents, 55% (49/89) were male, and 64% (16/25) of the cases were male. Cases predominantly occurred among men in the age of 30–39 years (38%, 9/24). In the highly affected group C, the male to female ratio among all group members was 2:1, whereas among cases 2.7:1.

**Table 1:** Dates of visit, attack rates and laboratory-confirmed cases by groups, hotel X, Norway, March 2007

Group	Dates of visit	Response rate %	Attack rate %	Number of laboratory- confirmed cases
Group A	12–16	65 (11/17)	0 (0/11)	0
Group B	15–16	38 (17/45)	12 (2/17)	0
Group C	16–18	75 (33/44)	67 (22*/33)	9
Group D	16–18	71 (10/14)	10 (1/10)	1
Group E	20–22	58 (7/12)	0 (0/7)	0
Group F	21–22	40 (4/10)	0 (0/4)	0
Group G	21–23	44 (7/16)	0 (0/7)	0

Total		56 (89/158)	28 (25/89)	10

* Of these 22, 14 were Swedish

**Figure 1:**
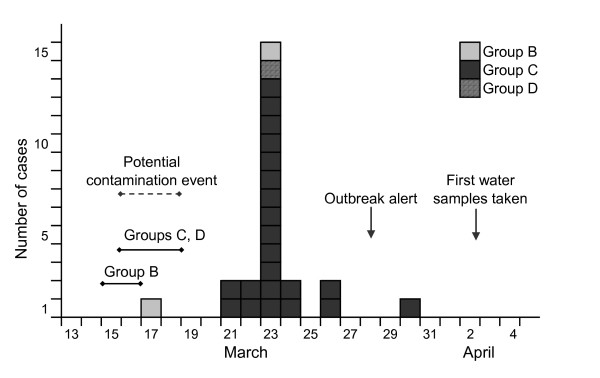
**Cases of gastroenteritis by day of disease onset, hotel X, Norway, March 2007 (n = 25)**. The figure indicates the time period when the contamination event might have occurred (dashed arrow) and the hotel stay period of the affected groups (solid lines). Arrow marks the day when the outbreak alert was sent from Sweden to Norway and the day when the first water samples were taken at hotel X.

Symptoms were reported from the first case on 17 March 2007. From 21 March, the number of cases increased and peaked on 23 March (figure 1). After the peak of the outbreak, only a few people reported symptoms. No respondent reported that they had travelled to regions considered risk areas for *Cryptosporidium *infection during the incubation period. Seven people in four groups (A, B, D and G) reported visiting the swimming pool at the hotel, but none of these were cases. Three cases (13%) reported that household members had developed similar symptoms after the cases returned from hotel X.

#### Clinical presentation

The most common symptoms reported in cases of laboratory-confirmed cryptosporidiosis were diarrhoea, abdominal cramps, and headache (table 2). Non-laboratory-confirmed cases reported similar symptoms. The median number of diarrhoeal episodes per day and median duration of illness was similar in cases with and without laboratory-confirmation (six and five episodes, and 12.5 and 10 days, respectively).

**Table 2:** Clinical symptoms by laboratory-confirmation of cryptosporidiosis in gastroenteritis cases, hotel X, Norway, March 2007

Symptoms	Lab-confirmed infection %	Non-confirmed infection* %	Total %
Diarrhoea	100 (9/9)	100 (15/15)	100 (24/24)
Bloody diarrhoea	0 (0/3)	0 (0/9)	0 (0/12)
Abdominal cramps	100 (9/9)	79 (11/14)	87 (20/23)
Headache	83 (5/6)	54 (7/13)	63 (12/19)
Fever	67 (4/6)	55 (6/11)	59 (10/17)
Nausea	75 (3/4)	45 (5/11)	53 (8/15)
Vomiting	25 (1/4)	9 (1/11)	13 (2/15)

Cases with missing or uncertain answer were excluded

* Negative stool sample or stool sample not taken

Seven of the 14 Swedish cases contacted a physician because of the symptoms, and doctors requested stool samples from five of these cases. None of the 11 Norwegian cases visited a physician except when requested as part of the investigation.

#### Analytical study

Based on the group-specific attack rates, laboratory-confirmed cases and dates of visit; the risk factor analysis was restricted to groups C and D. In analysing group C separately, the attack rate was higher among those who consumed water from hotel dispensers, tap water, broccoli soup, and lower among those who drank commercial bottled water (table 3). In combined analysis of groups C and D together, consumption of ice cubes in the bar was a strong risk factor, and the association between eating broccoli soup and illness remained. The attack rate was lower among those who reported eating fruit.

**Table 3:** Risk factors for gastroenteritis in groups C and D, hotel X, Norway, March 2007

	Exposed		Non-exposed		RR	95% CI	p value (2-sided Fisher's exact)	Percentage of cases exposed
					
	n/N	AR (%)	n/N	AR (%)				
*Group C (n = 22, N = 33)*								
Water from dispenser^†^	18/21	(85)	2/7	(29)	3.0	0.9–9.8	<0.01	90
Ice cubes from the bar	19/24	(79)	1/3	(33)	2.4	0.5–11.9	0.2	95
Tap water	14/16	(88)	3/8	(38)	2.3	0.9–5.8	0.02	82
Broccoli soup*	21/24	(88)	0/4	(0)	8	-	<0.01	100
Bottled water (commercial)	7/13	(54)	9/10	(90)	0.6	0.4–1.0	0.1	44
Fruit	9/14	(64)	6/7	(86)	0.8	0.5–1.2	0.3	60
*Combined Group C and D (n = 23, N = 43)*								
Water from dispenser^†^	18/28	(64)	2/7	(29)	2.2	0.7–7.5	0.1	90
Ice cubes from the bar	19/24	(79)	1/9	(11)	7.1	1.1–45.7	<0.001	95
Tap water	14/22	(64)	3/10	(30)	2.1	0.8–5.8	0.1	82
Broccoli soup*	22/32	(69)	0/4	(0)	8	-	0.02	100
Bottled water (commercial)	7/15	(47)	9/15	(60)	0.8	0.4–1.5	0.5	44
Fruit	9/22	(41)	6/7	(86)	0.5	0.3–0.9	0.1	60

n, case; N, total; AR, attack rate; RR, risk ratio; CI, confidence interval

* Part of menu on 16 March; ^† ^Different water dispensers were used by different groups

Broccoli soup was part of the menu served on 16 March, thus persons in four groups (A, B, C, and D) might have consumed it. For selected exposure variables, we calculated percentage of persons exposed in each group (table 4). Exposure to ice cubes occurred predominantly in the highly affected group C (88%), in contrast with low ice cube consumption in the other groups (10–25%). Other risk exposures did not differ considerably across groups A, B, C, and D. The proportion of persons who consumed, or possibly consumed, broccoli soup was high in each of the four groups (78–100%).

**Table 4:** Percentage of persons exposed to selected variables in four groups, hotel X, Norway, March 2007

	Percentage of persons exposed^‡ ^per group			
	
Exposure	Group A	Group B	Group C	Group D
Ice cubes in the bar	25	10	**88**	18
Drinking water bottled at hotel	75	70	64	18
Water from hotel dispenser^†^	56	100	73	73
Bottled water (commercial)	44	30	67	55
Tap water	63	60	71	20
Water as usual drink at meals	100	100	85	70
Broccoli soup*	80	100	85	78
Beef filet au gratin*	80	90	88	89
Grilled polenta*	87	80	75	78
Baked apple*	93	100	85	44
Zabaglione sauce*	80	100	61	67

‡ Answered "yes" or "possibly" consumed; † Different water dispensers were used by different groups

* Part of menu on 16 March

### Clinical microbiological investigation

Altogether 16 Swedish persons submitted stool samples, and stool examinations were positive for *Cryptosporidium *in 9 cases. Species-specific typing verified *C. parvum *in the Swedish isolates. Of the 4 Norwegian cases who submitted samples, one individual was positive for cryptosporidiosis.

### Environmental investigation

The hotel is located in a municipality with approximately 50,000 inhabitants, and is connected to the water distribution network of the local water works which supplies 100,000 people in that area and in a neighbouring municipality. The supply is based on a surface water source and the water treatment at the time was chlorination with no filtration. Water samples taken at the hotel and in the local water supply area in the beginning of April 2007 showed no evidence of contamination by *Cryptosporidium *and *Giardia*. Two oocysts were detected in a ten-litre sample of raw water and one presumptive oocyst was found in a ten-litre sample of treated water taken from the distribution system four months after the outbreak, in mid-June 2007.

Prior to the outbreak, the last water sample analysed for parasites was taken in November 2006, and was negative. Routine water samples taken on 19 March 2007 by the municipal waterworks showed no presence of *E. coli *or coliform bacteria in the local water supply.

Several tap water appliances were identified in the hotel (table 5), including two ice machines; one with a filter which was in the meeting rooms' kitchen and one in the bar which did not have a filter. Six meeting rooms had water dispensers directly connected to the water network (water samples were collected from dispensers at two meeting rooms which had served groups A, C, and D). Although the hotel was responsible for the maintenance of the ice machine placed in the meeting rooms' kitchen, no routine had been developed for this purpose. The filter of this ice machine had not been changed for more than a year over the recommended time. The filter was found to be very dirty at analysis which encumbered the laboratory examination for parasites, and only 5% of the filter was tested. The result was negative for *Cryptosporidium*. Filters in water dispensers could not be analysed as they had been changed as part of the hotel routine procedures shortly after the environmental investigation started.

**Table 5:** Tap water appliances, hotel X, Norway, March 2007

Tap water appliances	Use	Filter	Time since last filter change*
Ice machine in a small supplementary kitchen (meeting rooms' kitchen)	Ice cubes for water jugs in meeting rooms	5 μm cellulose filter (Cuno Micro-Klean II, G78 B2)	18 months
Ice machine in the bar	Ice cubes for drinks in the bar	Directly connected to water pipe	NA
Water dispensers in meeting rooms	Drinking water in meeting rooms	5 μm carbon filter (Omnipure K 2520P) and <0.5 μm ceramic filter (NSF standard 42&53)	5.5 months
Water purification system in the main kitchen	Bottled drinking water in the dining hall	1 μm particle filter and carbon filter, and an UV filter	Changed 2–3 times a year^†^

NA, non applicable; NSF, National Sanitation Foundation; UV, ultraviolet

* Recommended time to replace filters is generally 6–7 months

† No controlled documentation available

On-site investigation and interviews revealed that one kitchen worker had vomited at home during the night of 14 March, following a meal at a fast food restaurant with four friends, of which three became sick. He was away from work on 15 March, and felt better afterwards. He had continued working in the hotel from 16 March. *Cryptosporidium *was not detected in his stool sample taken in early April.

The food sample of raw beef carpaccio examined for common bacterial pathogens was of good hygienic quality.

## Discussion

We describe an outbreak of cryptosporidiosis among visitors to a Norwegian conference hotel in March 2007. Although the specific vehicle of the outbreak was not definitively identified, the results of the epidemiological and environmental investigation suggest that it may have been contaminated tap water through the ice machine in the bar and/or a water dispenser. It is notable that this outbreak would probably have remained undetected in Norway if it had not been for the alert from the Swedish health authorities. Despite frequent diarrhoeal episodes and prolonged length of illness, only one third of cases visited their doctors after their symptoms started, and none of these were Norwegians.

Most of the cases (88%) occurred in one of the visiting groups. Considering the incubation period of cryptosporidiosis (1–12 days, average 7 days), the epidemic curve indicated that cases must have been infected between 16 and 18 March, when two groups were staying at the hotel. Consumption of water from a hotel dispenser was a risk factor for illness in separate analysis of the group with the highest attack rate, indicating the possibility that a contamination event may have occurred in the water dispenser at their meeting room. In the two groups merged, those who had ice cubes in drinks from the bar were seven times more likely to develop illness than those who did not. Consumption of ice cubes with drinks in the bar was very common in the highly affected group, but infrequent in all the others. Ice cubes are possible vehicles of cryptosporidiosis as oocysts may survive freezing [11-14]. The one laboratory-confirmed case in the other group was the only person in that group who reported drinking more than five glasses of water from dispensers as well as from the tap. Also, this individual recalled drinking water at different places in the hotel, not only in their meeting room, and it is possible that the person had consumed water from the dispenser in the same meeting room as the group with the high attack rate.

In this outbreak, an exact mechanism of contamination has not been found. Contamination of ice cubes by bar personnel or by guests is unlikely, as the ice cubes were added to the drinks using a scoop. The ice machine in the bar had no filter, it was directly connected to the water system, thus *Cryptosporidium *oocysts, which may have been temporarily present in the treated water, could get into ice cubes. The water dispensers were equipped with carbon filters of 5 μm and ceramic filter of <0.5 μm pore size. Such a filtration system would be effective at removing oocysts; nevertheless, the filter pores could have been clogged by sediments or particles resulting in a filter breakthrough, and thus oocysts could possibly get into the water tank of a dispenser. Additionally, the attack rate was lower among those who consumed commercial bottled water. Point-of-use contamination of water dispensers and ice cube machines has been previously implicated in gastroenteritis outbreaks due to parasites [15,16]. Use of water dispensers, as well as ice cube machines, is very widespread in facilities such as hotels, offices, universities and hospitals. Appropriate cleaning and filter replacement procedures at the recommended intervals are important for preventing microbial contamination. As a minimum, ice cube machines should be completely sanitized on an annual basis, furthermore housekeeping duties should include regular cleaning of these units.

Several alternative routes of transmission were discussed. One person reported symptoms six days before the majority of cases occurred. We considered it unlikely that this person was the source of the outbreak since only one other person was infected in this group, and they had only one overlapping day with the group with most of the cases. The broccoli soup showed a strong association with illness in the epidemiological analysis. However, persons in four of the groups were exposed to the soup, and had the soup been contaminated with *Cryptosporidium*, we would have expected high attack rates in all four groups. The soup had been cooked for 30 minutes which is sufficient to kill oocysts, nonetheless croutons were added manually when served. The kitchen worker, with symptoms of food intoxication, was not involved in the handling of the soup, and his subsequent stool sample was also found to be negative for parasites. Nevertheless, kitchen workers should wait for at least 24 hours after their gastrointestinal symptoms stop before returning to work. The importance of clean hands, i.e. maintaining a good standard of hygiene when handling food and drinks, and when using the bathroom facilities cannot be emphasised enough in preventing the development and spread of infections; particularly in relatively closed settings like conference hotels where a large number of persons may reside.

Environmental samples, collected two weeks after the probable contamination event was thought to have occurred, showed no presence of the parasite. Nonetheless, the raw water sample found positive for *Cryptosporidium *in mid-June may indicate a fluctuating occurrence of low concentrations of oocysts in the distribution area which may be at the time of a given sampling just below the detection limit of the water testing methods. It should be noted that low level contamination is not unusual and not necessarily an indication that oocysts numbers necessary for infection are not present. The number of *Cryptosporidium *oocysts needed to cause human infection can be as low as 9–10 oocysts (50% infectious dose) for both *C. parvum *and *C. hominis *[17,18], and oocysts are often not detected in cryptosporidiosis outbreaks associated with public drinking water [19]. This could be because samples were taken too late after the contamination event, or because the oocyst concentrations that triggered illness were too low to be determined.

In Norway, *Cryptosporidium *and *Giardia *have been identified in low concentrations in 19% of raw surface water sources sampled [20]. Additionally, *Cryptosporidium *positive samples of sewage influent were found in 32 of 40 examined sewage treatment works [21]. Removal of parasites like *Cryptosporidium *and *Giardia *from sewage influent is assumed to be minimal, since many sewage treatment works use primary treatment processes only [21]. Further contamination may occur when sewage treatment works discharge effluent containing parasites into natural waters. While filters or UV-equipment are necessary to remove or inactivate oocysts, it is common for Norwegian waterworks to have chlorination as the only water treatment [22].

At present, cryptosporidiosis – unless associated with diagnosis of AIDS – is not notifiable in Norway. Reporting of outbreaks and diagnosing cases with parasitic gastrointestinal diseases are limited, as awareness of parasitic pathogens is low among physicians, and laboratories do not routinely analyze faecal samples for *Cryptosporidium *or *Giardia*. Both these factors contributed to the late detection of a large waterborne outbreak of giardiasis in Norway in 2004 [23]. Ten of the 14 medical microbiological laboratories with available diagnostics for *Cryptosporidium *reported less than 10 examinations requested per year [4]. Among the patients who contacted their physicians in Sweden, in many cases stool samples were taken for analysis for parasites. Prompt reporting of these unusual cases to the Swedish public health authorities led to the relatively early recognition of the outbreak.

Interestingly, as none of the Norwegian cases sought medical advice from their physicians in this case, the lack of routine laboratory analysis for parasites in patient samples cannot be considered to have contributed to the lack of detection of this outbreak in Norway. However, it is also unlikely that elevated immunity to cryptosporidiosis amongst the Norwegian hotel guests contributed to their lack of contacting a physician as a similar spectrum of symptoms were reported amongst both Swedish and Norwegian hotel guests upon questioning; the difference in post-symptom behaviour between Swedish and Norwegian hotel guests can be speculatively assigned to an unidentified cultural difference. Whether the lack of reported *Cryptosporidium *infection amongst residents of the municipality served by the same water supply is indicative of enhanced immunity to this infection in this community, as previously described by Frost et al. [24], or merely a reflection of the hotel residents receiving a larger, or otherwise more infectious, dose of the infectious agent, could not be determined. Certainly serological study of this community might provide interesting insights. Nevertheless, this outbreak highlights the importance of encouraging physicians to submit samples for parasitological examination from patients with symptoms consistent with such infections, even if they have not travelled abroad or had animal contacts, and to ask patients about other people they know experiencing similar gastrointestinal symptoms.

## Conclusion

We have described the first laboratory-confirmed human cryptosporidiosis outbreak in Norway probably linked to water. The lessons in the investigation are diverse and the issues described regarding the diagnosis of parasitic diseases may not be limited to the country. Cryptosporidiosis is among the communicable diseases that are notifiable at European Union-level; however, it is currently among the diseases with least surveillance in Europe [25,26]. For the future, introduction of cryptosporidiosis into the national reporting system in Norway should be considered. This would primarily be useful in increasing awareness and improving diagnosis of parasitic gastrointestinal infections, and may also improve outbreak detection; furthermore, it would enable better understanding of the epidemiology and public health burden of the disease in the country.

## Competing interests

The authors declare that they have no competing interests.

## Authors' contributions

AgH, LV, KN were responsible for the epidemiological investigation including the cohort study and AgH drafted the manuscript. TAØ, AnH, SRH were responsible for the local epidemiological and environmental investigations of the outbreak. TK contributed significantly to the interpretation of the epidemiological as well as environmental results. LR carried out microbiological investigations including the examination of the cellulose filter. BdJ notified the Swedes cases to Norway and coordinated the outbreak investigation of the Swedish part. All authors critically revised the article for important intellectual content, and all authors have seen and approved the final version.

## Pre-publication history

The pre-publication history for this paper can be accessed here:


